# Video-assisted Thoracoscopic Resection of a Giant Bulla in Vanishing Lung Syndrome: case report and a short literature review

**DOI:** 10.1186/1749-8090-9-4

**Published:** 2014-01-05

**Authors:** Kobe Van Bael, Mark La Meir, Hans Vanoverbeke

**Affiliations:** 1 Department of Cardiothoracic Surgery, ASZ Aalst, Merestraat 80, B-9300 Aalst, Belgium

**Keywords:** Giant bulla, *Pulmonary emphysema*, Vanishing lung syndrome, *Thoracoscopic resection*, *Bullectomy*, *VATS*

## Abstract

A 36-year-old Caucasian man was admitted to our hospital with acute onset of left-sided chest pain. Computed Tomography confirmed the presence of a giant bulla on the apex of the lower lobe of the left lung. A video-assisted thoracic surgery (VATS) with bullectomy was performed using two linear endostaplers. Additionally pleurectomy was performed. No serious complications occurred in the postoperative course, as the patient showed good lung re-expansion and no prolonged air leakage.

VATS bullectomy is a suitable and eminent technique to approach giant bullous emphysema and definitely fulfils a role in its treatment.

## Background

Giant bullous emphysema (GBE) involves the presence of emphysematous areas with complete destruction of lung tissue producing an airspace bigger than 1cm in diameter. An anatomical classification is made based on single or multiple bullae and the absence or presence of diffuse emphysema [1]. Obviously the bullous area does not participate in broncho-alveolar oxygenation and can cause dyspnoea, hypoxia, symptomatic chest pain or pressure, haemoptysis etc. It can result in spontaneous pneumothorax, pneumothorax provoked by mechanical ventilation, infection and even slow progression to malignancy.

Some literature differentiates GBE from bullous emphysema where the latter has been associated with more diffusely abnormal lung tissue (in the context of chronic obstructive pulmonary disease) and where GBE has bullae with structural normal intervening lung parenchyma.

GBE, sometimes referred to as Vanishing Lung Syndrome (VLS) as a clinical syndrome, was first described by Burke in a typical patient: a young male cigarette smoker with a large bullae in the upper lobe associated with paraseptal emphysema [2]. Roberts described radiographic criteria for this entity: the presence of a giant bullae in one or more upper lobes (mostly unilateral), often asymmetrical, occupying at least one-third of the hemithorax and compressing surrounding normal lung parenchyma [3].

High resolution computerized tomography (HRCT) is the best imaging technique to determine most accurately the extent and distribution of bullous disease. CT also allows assessment of coexisting problems such as bronchiectasis, co-infected cysts, pulmonary artery enlargement and pneumothorax [4,5]. Centrilobular emphysema is mostly seen on HRCT in cigarette smokers and can be of major importance pre-operatively.

The condition has clearly been associated with smokers, alpha-1 antitrypsin deficiency and marijuana abuse [6]. Some reports also mention Marfan and Ehlers-Danlos syndrome as possible causes.

Surgery is indicated to treat the complications related to GBE or on preventive basis when lesions occupy more than one third of the hemithorax, when there is a compression of healthy adjacent lung tissue and when size of a bulla shows to have been increased at follow-up. Generally resection of small bullae has no effect on lung function [7,8]. Also auto-bullectomy has been reported [9].

The case described below illustrates the successful approach of a bullectomy via VATS for GBE.

## Case presentation

A 36-year-old Caucasian male patient was admitted to the emergency department with acute onset of left thoracic chest pain. The pain suddenly appeared without physical activity, cough or trauma. Medical history of this patient revealed a chronic low back pain, surgery for left epicondylitis lateralis humeri and no specific cardiovascular risk factors except for active smoking.

Physical examination showed a man with diminished breath sounds on the left apex over the left anterior chest without palpable subcutaneous emphysema and with normal oxygen saturation. There were no other abnormal clinical findings.

**Figure 1:**
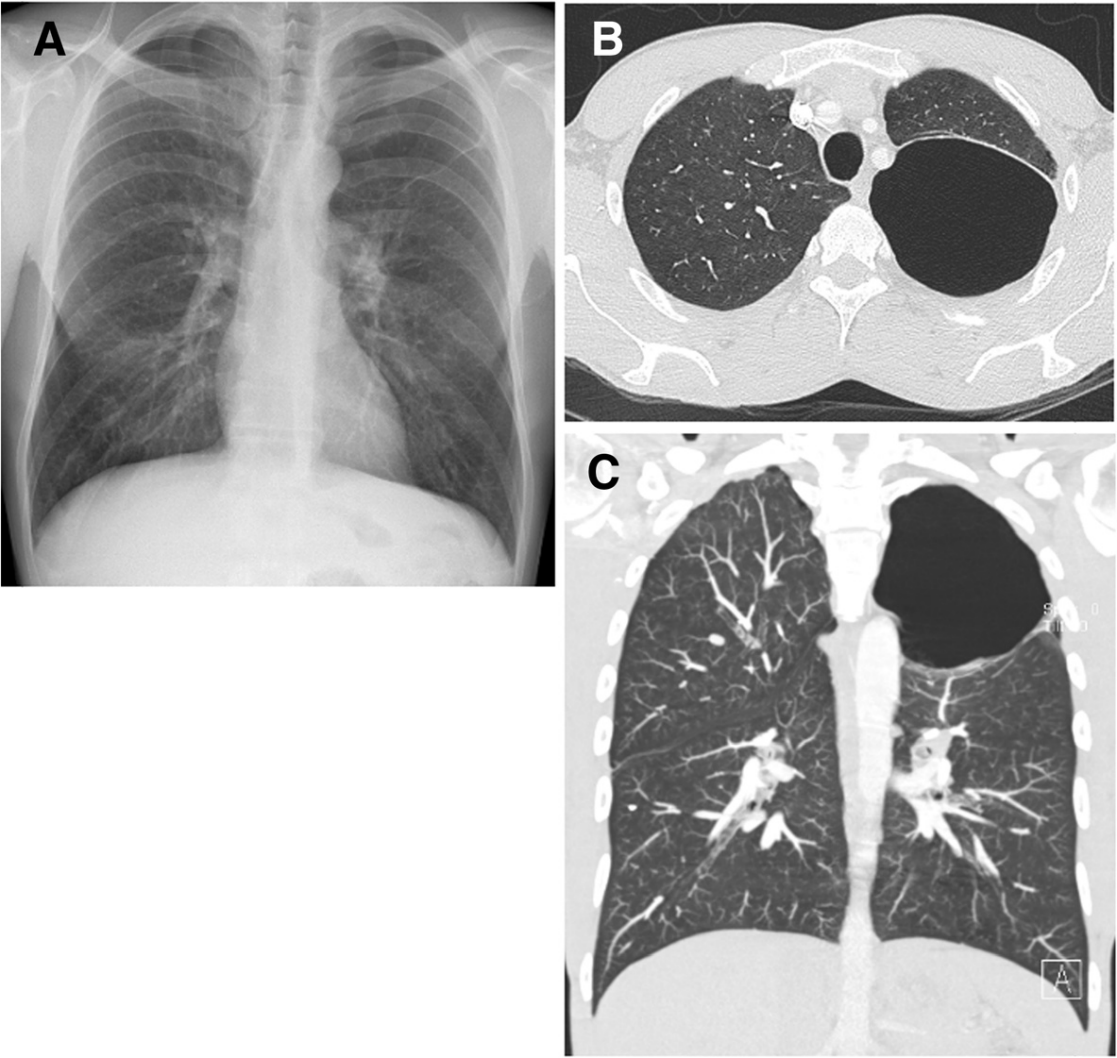
**Initial chest radiograph of the patient.** (PA view) showing a giant bulla (increased hyperlucent zone) of the left apex **(A)**. HRCT showing axial **(B)** and sagittal **(C)** image (lung window) with a big lucent area without real definable walls.

A routine chest X-ray was performed and suggested an apical pneumothorax on the left side, though HRCT showed a massive bulla of the left lung, with a 10 cm diameter, occupying the whole upper left hemithorax, with signs of centrilobular emphysema also on the right side [Figure 1A-C].

The initial arterial blood gas analysis without oxygen showed pH 7.38, pO2 94 mmHg, and pCO2 43 mmHg. Carboxyhemoglobine status was 6.4% and other blood results were normal.

**Figure 2:**
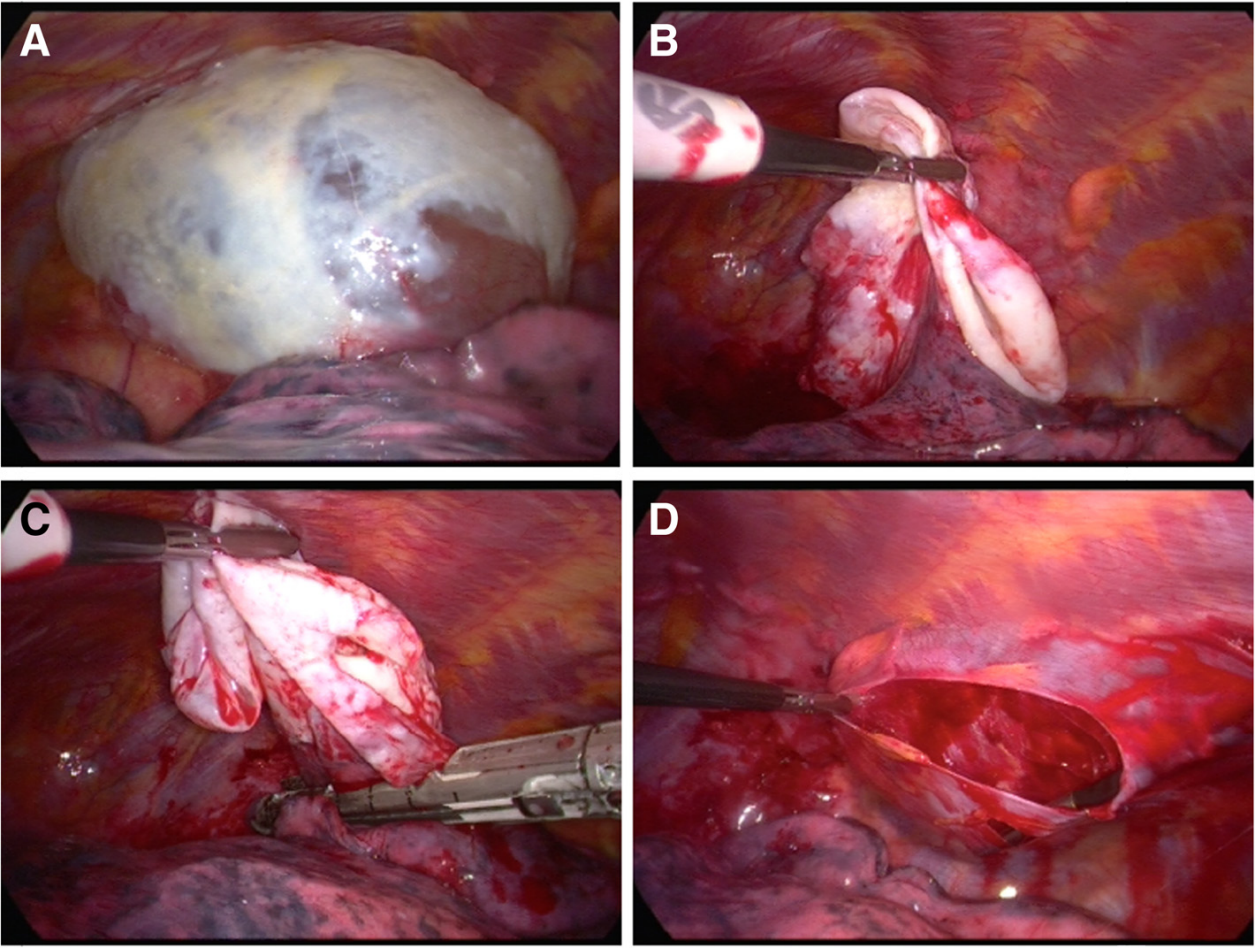
**Intra-operative VATS-image of the giant bulla.** Visualisation of bulla **(A)**, local resection **(B & C)** and pleurectomy **(D)**.

The patient underwent VATS with bullectomy. Surgery was performed under general anaesthesia with double lumen endotracheal intubation and discontinuing ventilation on the left side in half lateral position. Two 12 mm trocars and one 5 mm trocar were used. The giant bulla was located at the apex of the lower lobe with total compression of the left upper lobe and pleural irritation [Figure 2A]. Bullectomy was done with two 60 mm Endo-GIA (Gastro-Intestinal Anastomosis) linear endoscopic stapling devices (Covidien®, Norwalk, CT, USA) without extra suture reinforcement [Figure 2B and C]. Subsequently an extensive stripping of the parietal pleura was carried out [Figure 2D].

Anatomopathologic examination of the pleural wall of the giant bulla showed signs of chronic inflammatory infiltrates without any malignancy.

The lung expanded well and the pleural cavity was drained with two chest tubes (thoracic drainage systems Pleur-evac) connected to a water seal under 10 cm H_2_0 suction. No air leak was noted, tubes were removed on second post-operative day.

Patient developed a bronchopneumonia of the right lower lobe, which was treated with intravenous antibiotics and quickly resolved. After five days the patient could leave the hospital in a good general condition.

Three months later the patient was free of complaints. Auscultation and spirometry were perfectly normal. No restrictive syndrome was observed with FEV1 of 78% (reversible), FVC of 82% and normal lung volumes.

**Figure 3:**
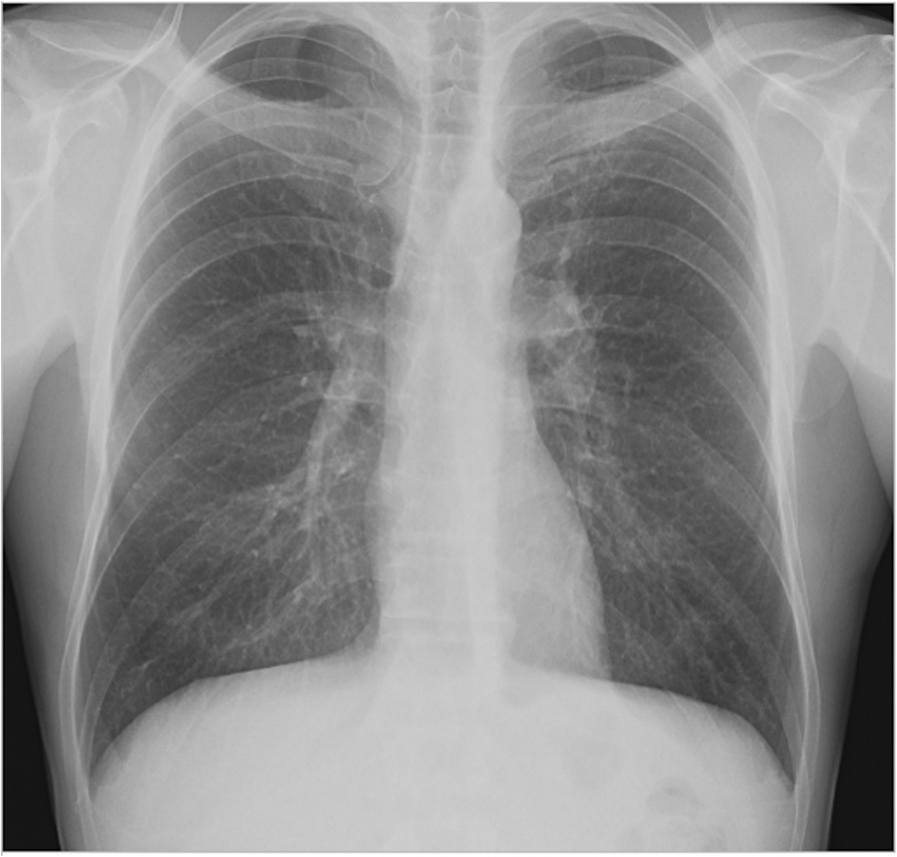
Follow up (post-operative) chest radiograph (PA view) of the patient showing a normal lung parenchyma of the left apex.

Chest radiography showed good expansion of the left lung without pneumothorax or residual pleural effusion [Figure 3]. The patient ceased tobacco consumption since surgery, supported by Vareniciline (Champix®, Pfizer, New York, USA). One year follow-up revealed no recurrence, no intercostal pain syndrome and preserved pulmonary function.

## Discussion

Surgery in GBE is a valid treatment option for a specific group of patients with emphysematous bullae who present symptoms like chest pain or spontaneous pneumothorax.

Different thoracoscopic techniques are reported in literature in the past, such as thoracoscopic bullous endoloop ligation, intra-cavitary bullous drainage, laser bullae ablation and bullous fibrin glue treatment, however all with variable success [10-13]. Almost all of the above techniques are now abandoned. A non-excisional treatment option is the Brompton technique, first described by Monaldi. In this technique the bulla is drained percutaneously and a mushroom Foley catheter is left behind after septectomy and talcage and is put under suction for a few days after which the bronchocutaneous fistula closes spontaneously after removal [14].

In the last decade the application of VATS bullectomy with endoscopic staple resection is considered as a suitable and safe treatment of choice [15].

Operative mortality rates after bullectomy for localised bullous emphysema in patients with preserved lung function are low (from 0 to 2.5%). Morbidity is primarily related to prolonged air leak (53%), atrial fibrillation (12%), postoperative mechanical ventilation (9%), pneumonia (5%) and postoperative incisional pain [16].

The few studies which report long-term follow-up of patients undergoing surgery for GBE show well documented significant early clinical and functional improvement that persists for at least 3 to 4 years but declines from then on to be almost totally reversed after 5 years in patients who present with underlying diffuse emphysema [8]. Long term outcome and survival data are rare in today’s literature.

Obviously overall mortality and morbidity is not so low in patients with compromised lung function. For these patients with heterogeneous emphysema safer alternative treatments as compared to classic lung volume reduction surgery (LVRS) are being considered. Transbronchial approach is another investigative technique [17].

Most authors feel that total resection of the bullae is of major importance since the risk of development of bronchogenic carcinoma is a lot higher in patients with GBE [18]. Venuta and Goldstein respectively reported an incidence of primary lung cancer associated with bulla as 4.2% and 3.8% [18,19]. The relative risk of lung cancer adjoining a pulmonary bulla is much higher, therefore requiring a strict follow-up. Differentiating malignancy in emphysematous lungs is a difficult diagnosis with HRCT, so low threshold towards biopsy or resection of growing low-density mass can be defended.

One of the most difficult post-operative complications is persistent air leakage which can prolong hospital stay significantly. Some groups use pericardial strips, PTFE meshes or other surgical sealants to prevent air leakage when stapling lung parenchyma in severely affected emphysematous lungs [20]. Other alternatives are partial pleurectomy to promote better pleurodesis but it can hamper a re-intervention in younger patients. We suggest to deflate the bulla and squeeze it between the endo-clamps so the base of the giant bulla becomes visible without injuring healthy lung tissue and possibly provoking air leaks. Performing bullectomy including a small strip of healthy lung tissue might help in the prevention of the above.

## Conclusion

### We presented a case of GBE that was successfully managed with VATS bullectomy

HRCT is essential in diagnosis and differentiation between localised and diffuse emphysema for careful pre-operative surgical assessment.

Accurate intra-operative management (by minimal invasive approach, proper endo-stapling, tube drainage and complete lung re-expansion) and adequate post-operative management (by early mobilisation, careful chest tube suction, good pain control and extensive physiotherapy) are vital to obtain a successful treatment with low morbidity and low mortality. Good selection remains a difficult and controversial subject.

In this patient’s case bullectomy by VATS approach for treatment of GBE proved to be safe and effective and led to an improvement in his quality of life, preventing further complication of the compressed lobe.

VATS should be the technique of choice, open surgery being obsolete in the treatment of GBE.

## Consent

Written informed consent was obtained from the patient for publication of this case report and any accompanying images. A copy of the written consent is available for review by the Editor-in-Chief of this journal.

## Abbreviations

VATS: Video-assisted thoracic surgery; GBE: Giant bullous emphysema; VLS: Vanishing lung syndrome; HRCT: High resolution computerized tomography; LVRS: Lung volume reduction surgery.

## Competing interests

No author has any commercial association that might create a conflict of interest in connection with this manuscript.

## Authors’ contribution

Dr. KVB drafted the manuscript and therefore made the major substantive contribution and was also assisting surgeon; Prof. MLM revised the manuscript for intellectual content and participated until the final approval of the submitted version; Dr. HV was the operating surgeon, coordinated the draft of the manuscript and revised the manuscript critically. All authors read and gave final approval for the version to be published.
